# Meta‐Analysis of the Relationship Between Occupational/Environmental Exposure to Wood Dust and Laryngeal Cancer

**DOI:** 10.1002/cam4.70330

**Published:** 2024-10-20

**Authors:** E. Meng, Zhou Xin, Dou Jianrui, Yin Jinzhu

**Affiliations:** ^1^ Yangzhou Center for Disease Control and Prevention Yangzhou Jiangsu China; ^2^ Shanxi Health Commission Key Laboratory of Nervous System Disease Prevention and Treatment Sinopharm Tongmei General Hospital Datong Shanxi China; ^3^ Datong Key Laboratory of Nervous Systems Disease Prevention and Treatment for Coal Mine Workers Sinopharm Tongmei General Hospital Datong Shanxi China; ^4^ Central Laboratory of Sinopharm Tongmei General Hospital Datong Shanxi China

**Keywords:** laryngeal cancer, meta‐analysis, wood dust

## Abstract

**Objective:**

Wood dust is a human carcinogen. However, studies examining the relationship between wood dust exposure and laryngeal cancer have yielded inconsistent findings. Therefore, we systematically reviewed relevant studies examining the relationship between wood dust exposure and laryngeal cancer development, followed by a meta‐analysis.

**Methods:**

Publications in the following databases were searched: PubMed, Medline, Embase, Cochrane Library, and China National Knowledge Infrastructure (CNKI). The Newcastle–Ottawa scale was used to evaluate the study quality. A random‐effects model was used for the meta‐analysis.

**Results:**

Eighteen case–control studies and one cohort study, involving a total of 4426 patients with laryngeal cancer and 319,129 control participants, were identified. The association between occupational/environmental exposure to wood dust and laryngeal cancer, if any, was unclear (adjusted combined OR: 1.11; 95% CI: 0.94–1.31). However, subgroup analyses according to the number of cases, geographic region, publication year, and follow‐up duration revealed correlations between wood dust exposure correlated and laryngeal cancer, as follows: number of cases > 200 (OR: 1.14; 95% CI: 1.01–1.25 [*n* = 10]); studies conducted in the US (OR: 1.21; 95% CI: 1.07–1.37 [*n* = 5]); follow‐up time > 5 years (OR: 1.19; 95% CI: 1.07–1.32 [*n* = 10]); and publication after the year 2000 (OR: 1.15; 95% CI: 1.04–1.28 [*n* = 8]). A high heterogeneity in the results was observed (*I* ^2^ = 42.5%, *p* = 0.024). The results were stable, and no publication bias existed, according to sensitivity analysis.

**Conclusions:**

This meta‐analysis suggests that wood dust exposure is associated with laryngeal cancer. Additional large‐scale studies are warranted to clarify the relationship between wood dust exposure and laryngeal cancer.

## 1 Introduction

Wood dust is a harmful by‐product widely present in wood‐processing industries such as sawmills, furniture manufacturing, and cupboard production [1]. The International Agency for Research on Cancer (IARC) classified wood dust as a class I carcinogen in 2009. Specifically, wood dust can cause nasal cavity, paranasal sinus, and nasopharynx cancer [2]. Notably, workers in a wide range of jobs are exposed to wood dust worldwide [3]. Approximately 1.7 million workers in the UK are exposed to low (< 0.5 mg/m^3^) or high (> 5 mg/m^3^) levels of wood dust [4]. Moreover, 2.3% of workers in Finland [5], 1.3% of workers in Italy [6], and 1.8% of workers in France [7] have been exposed to wood dust. The highest cumulative wood dust exposure is 28.82 mg/m^3^‐y in four Nordic countries, and many workers have been exposed to wood dust for > 10 years [8]. In France, approximately 75 cases of occupational disease after exposure to wood dust have been reported every year, and compensation of 20–30 million euros is paid to employees [9].

Exposure to wood dust can lead to several health problems, particularly respiratory tract cancer. A meta‐analysis has indicated that exposure to wood dust has a stronger association with nasal adenocarcinoma than lung cancer [10]. Our previous study confirmed that wood dust exposure poses a dose‐dependent risk of nasopharyngeal carcinoma [11] (OR: 2.18; 95% CI: 1.62–2.93). However, studies on the association between wood dust exposure and laryngeal cancer have shown widely varying results and have yielded no definitive findings regarding the association. Some studies have reported wood dust exposure as a potential risk factor for laryngeal cancer [12, 13, 14, 15, 16, 17, 18, 19, 20], some have demonstrated an association [21, 22, 23], whereas others have found no association [24, 25, 26, 27, 28, 29, 30]. A recent meta‐analysis by Paget‐Bailly, Cyr, and Luce [31] has concluded no association between wood dust exposure and the risk of laryngeal cancer. Notably, that meta‐analysis [31] included studies published until 2012, and the association between wood dust exposure and laryngeal cancer in some studies was analyzed according to the standardized mortality ratio. Many cases included in these studies were not original cases, and the sample sizes were small. The populations in Wortley's et al. 1992 study [24] and Vaughan and Davis 1991 study [32] were from the same case–control study. Because the evidence linking wood dust exposure to laryngeal cancer risk is inconclusive, the objective of the current meta‐analysis was to increase the study sample size and to investigate the potential association, if any, through a case–control design.

## 2 Materials and Methods

This meta‐analysis was based strictly on the Preferred Reporting Items for Systematic Reviews and Meta‐Analysis (PRISMA) statement [33] and Cochrane Collaboration guidelines [34].

### 2.1 Search Strategy

We searched the PubMed, Embase, Medline, Cochrane Library, and China National Knowledge Infrastructure (CNKI) databases without language restrictions. The search for original manuscripts was conducted on database records from January 1991 through June 2024. To search for additional studies, we also manually searched the references of the included studies.

Search strings included the following indexing terms: wood dust (“wood dust,” “sawdust,” “saw dust,” “hardwood dust,” and “softwood dust”); and laryngeal cancer (“laryngeal cancer,” “larynx cancer,” “laryngeal carcinoma,” and “laryngeal malignancy”). No additional restrictions were used in the search.

### 2.2 Study Selection and Eligibility Criteria

The inclusion criteria were as follows: (1) cohort or case–control study design; (2) reporting of the role of occupational/environmental exposure to wood dust in laryngeal cancer, including the effect size (odds ratio [OR] and relative risk) and confidence interval (CI), with adjustment for confounding factors; (3) control group comprising individuals with low or no exposure to wood dust; (4) confirmation of laryngeal cancer through clinical diagnosis in a hospital (International Classification of Diseases for Oncology [ICD‐O]); and (5) original reports.

The exclusion criteria were as follows: (1) studies not pertaining to wood dust exposure and laryngeal cancer; (2) studies with insufficient data; (3) case reports, reviews, and commentaries; and (4) cross‐sectional studies.

Additionally, only studies with relatively large sample sizes were selected to ensure study independence if the data were used in more than one study or if one study included findings from multiple studies.

### 2.3 Data Extraction

Two reviewers (Meng and Jinzhu) independently assessed the titles and abstracts of the selected studies and compared the data separately to ensure data completeness and accuracy. The following data were extracted: author; date of publication; study year(s); study type; number of cases and controls; geographic location where the study was conducted; OR; 95% CI; and adjustment factors. Any inconsistencies in data extraction were resolved through discussion with a third reviewer.

### 2.4 Quality Assessment

The quality of the selected studies was assessed with the Newcastle–Ottawa scale [35], comprising nine items, each assigned a value of 1 point. The study quality assessment was independently performed by two authors, and inconsistent scores were resolved through discussion. Only scores ≥ 5 were selected on the basis of a previous study [11].

### 2.5 Statistical Analysis

Statistical analysis was performed in STATA 14.0 (Stata Corp LP, city, state, country). The combined OR and 95% CI were calculated, and the *Z*‐test was used to determine whether the combined OR values were statistically significant at *p* < 0.05. Cochran's *Q* test, quantified by the *I* ^2^ statistic [36], was applied to assess the heterogeneity. A random‐effects model was used to calculate the combined OR for *I* ^2^ > 50%. Begg's and Egger's tests were used to detect the publication bias (with *p* < 0.05 considered statistically significant). Sensitivity analysis was performed with a leave‐one‐out design to assess the study stability.

## 3 Results

### 3.1 Study Selection

**FIGURE 1:**
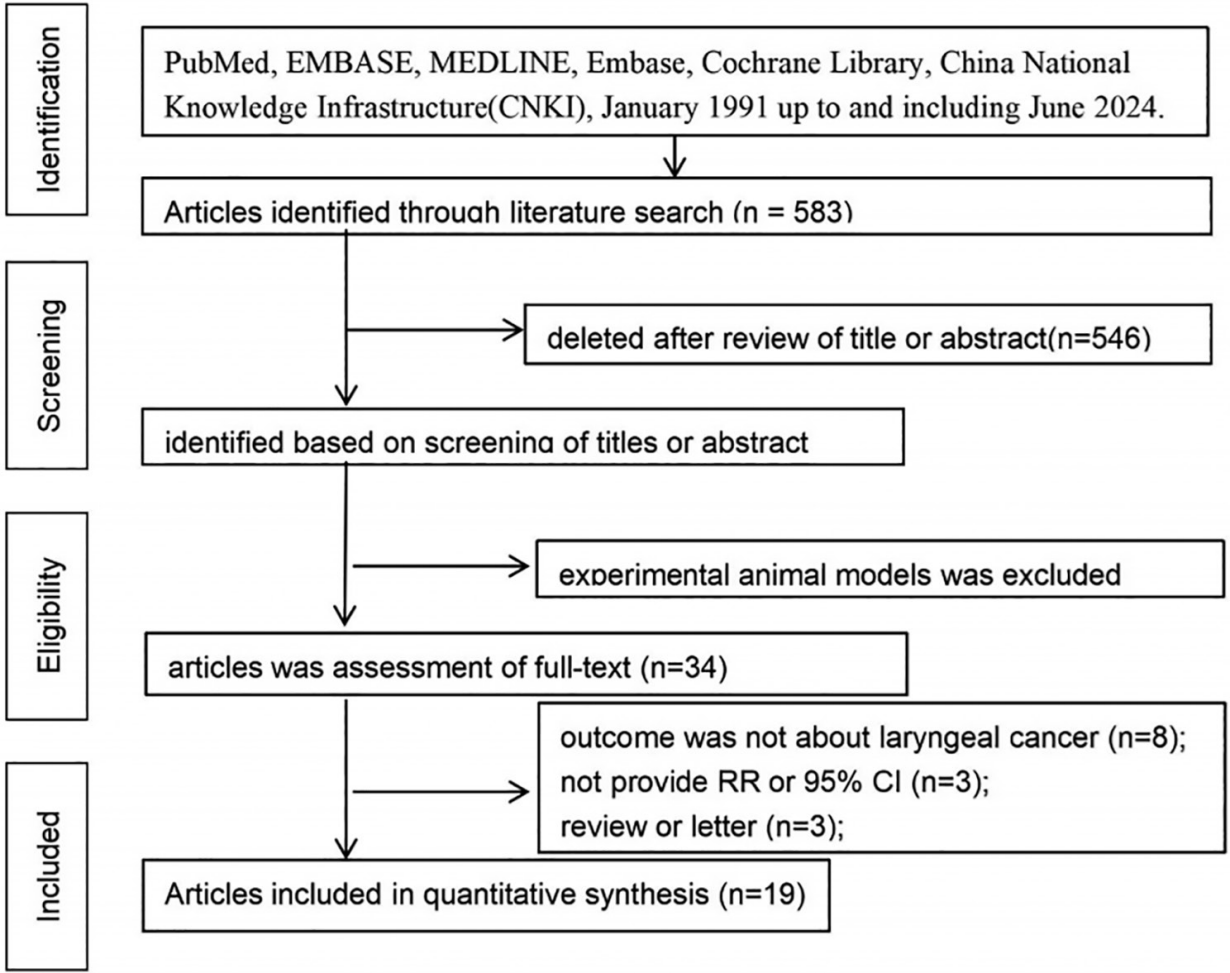
Preferred reporting items for systematic reviews and meta‐analyses (PRISMA) flow diagram showing the retrieval process of studies included in the systematic review.

As shown in Figure 1, 583 studies were screened during the initial search, among which 548 did not meet the inclusion criteria for content, study design, target population, and outcomes and were excluded according to the titles and abstracts. The remaining 33 studies were completely and carefully reviewed. The current study included 18 case–control studies and one cohort study. Three studies were from Germany [11, 13, 14]; five were from the US [6, 8, 9, 12, 15]; and one each were from Turkey [3], Estonia [4], France [5], China [7], Uruguay [10], New Zealand [17], Sweden [18], Switzerland [19], and Spain [22]. The 2003 Berrinol study [25] included studies from France, Italy, Spain, and Switzerland. The 2006 Shangina study [20] included studies from Poland, Romania, Russia, and Slovakia (Table 1).

**TABLE 1:** Occupational/environmental exposure to wood dust and risk of laryngeal cancer included in the study.

No.	Author (years)	Study	Country (city)	Follow year	Case	Control	RR/OR	95% CI		Adjustment for confounding factors
1	Elci OC (2002) [12]	Case–control study	Turkey	1979–1984	940	1519	1.1	0.8	1.4	Age, smoking, drinking
2	Jayaprakash V (2008) [13]	Case–control study	Estonia	1982–1998	124	1522	1.52	0.79	2.86	Age, education, smoking
3	Laforest L (2000) [14]	Case–control study	France	1989–1991	296	296	1	0.58	1.72	Age, smoking, drinking
4	Muscat JE (1991) [15]	Case–control study	USA (New York)	1985–1990	194	184	1.7	0.7	4.6	Age, education, drinking
5	Zheng W (1992) [16]	Case–control study	China	1989–1990	201	414	1.4	0.6	3.2	Age, smoking
6	Zagraniski RT (1986) [17]	Case–control study	USA (New Haven)	1975–1980	87	153	2.5	0.5	12.9	Smoking, drinking
7	Brown (1998) [18]	Case–control study	USA (Texas)	1975–1980	183	250	1.51	0.86	2.67	Age, smoking, drinking
8	De Stefani (1998) [19]	Case–control study	Uruguay	1993–1995	112	509	1.6	0.6	4.2	Age, environment, education, smoking, drinking
9	Ramroth H (2008) [20]	Case–control study	Germany	1998–2000	257	769	2.1	1.2	3.9	Smoking, drinking, education
10	Langevin SM (2013) [21]	Case–control study	USA (Boston)	2006–2011	213	1442	1.2	1	1.3	Age, gender, race, smoking, drinking, education, HPV16
11	Maier MH (1997) [22]	Case–control study	Germany (Heidelberg)	1988–1989	164	656	2	1.1	3.9	Smoking, drinking
12	Ahrens W (1991) [23]	Case–control study	Germany (Bremen)	1986–1987	85	100	0.7	0.2	2.2	Age, smoking, drinking
13	Wortley P (1992) [24]	Case–control study	USA (Washington)	1983–1987	235	547	0.5	0.2	1.5	Age, smoking, drinking, education
14	Berrino F (2003) [25]	Case–control study	France /Italy/Spain/Switzerland	1979–1982	213	819	0.6	0.3	1.3	Age, area, drinking, income
15	Kawachi I (1989) [26]	Case–control study	New Zealand	1980–1984	379	735	0.79	0.43	1.46	Age
16	Purdue MP (2006) [27]	Cohort study	Sweden	1971–2001	227	307,799	0.8	0.50	1.5	Age, smoking
17	Gustavsson P (1998) [28]	Case–control study	Switzerland	1988–1990	157	641	0.54	0.32	0.93	Age, smoking, drinking
18	Shangina O (2006) [29]	Case–control study	Poland, Romania, Russia, Slovakia	1999–2002	316	728	0.81	0.48	1.37	Age, country, smoking, alcohol
19	Pollan (1995) [30]	Case–control study	Spain	1982–1985	43	46	2.69	0.94	7.67	Smoking, drinking

### 3.2 Meta‐Analysis of Wood Dust Exposure and Laryngeal Cancer

**FIGURE 2:**
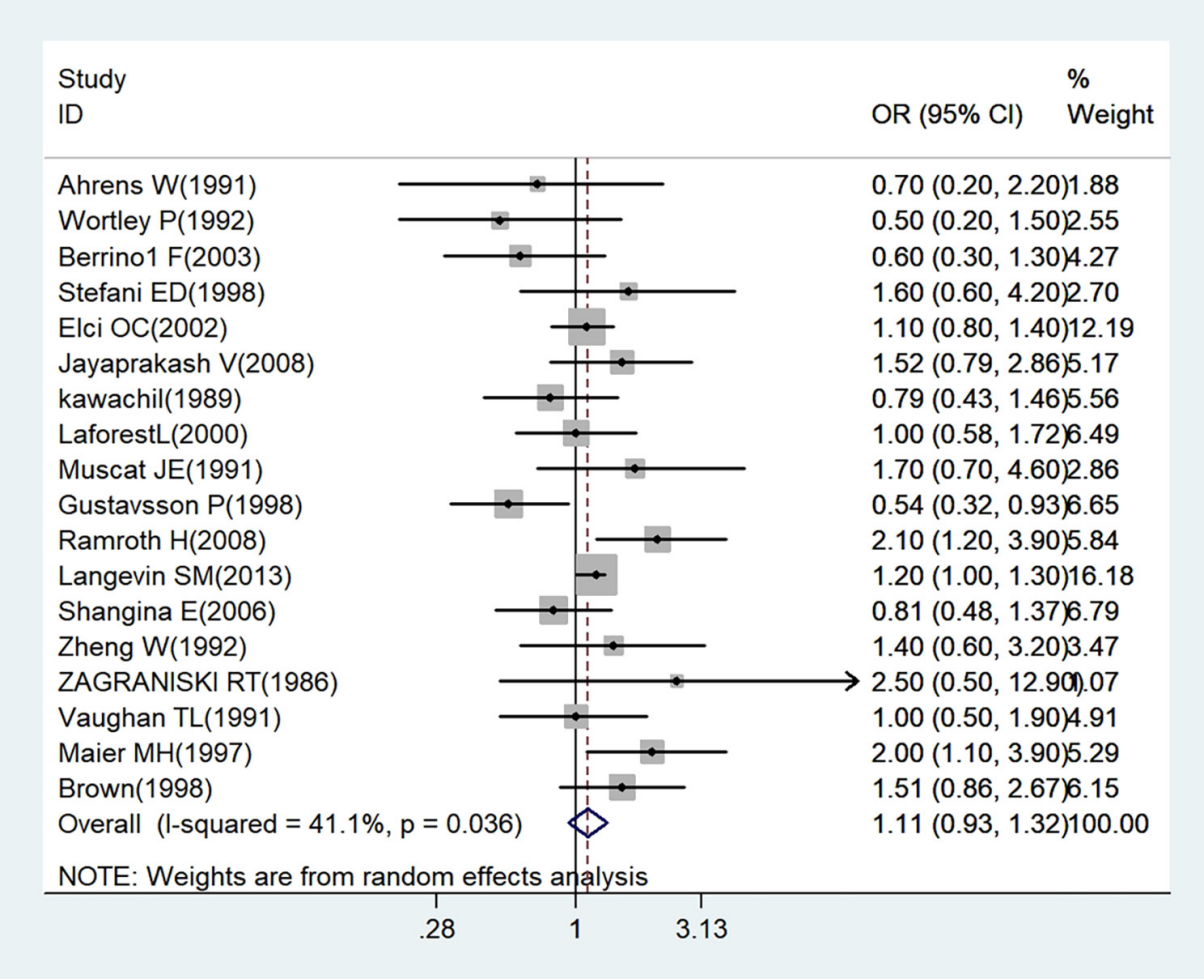
Forest plot for meta‐analysis of the relationship between occupational/environmental exposure to wood dust and laryngeal cancer.

**FIGURE 3:**
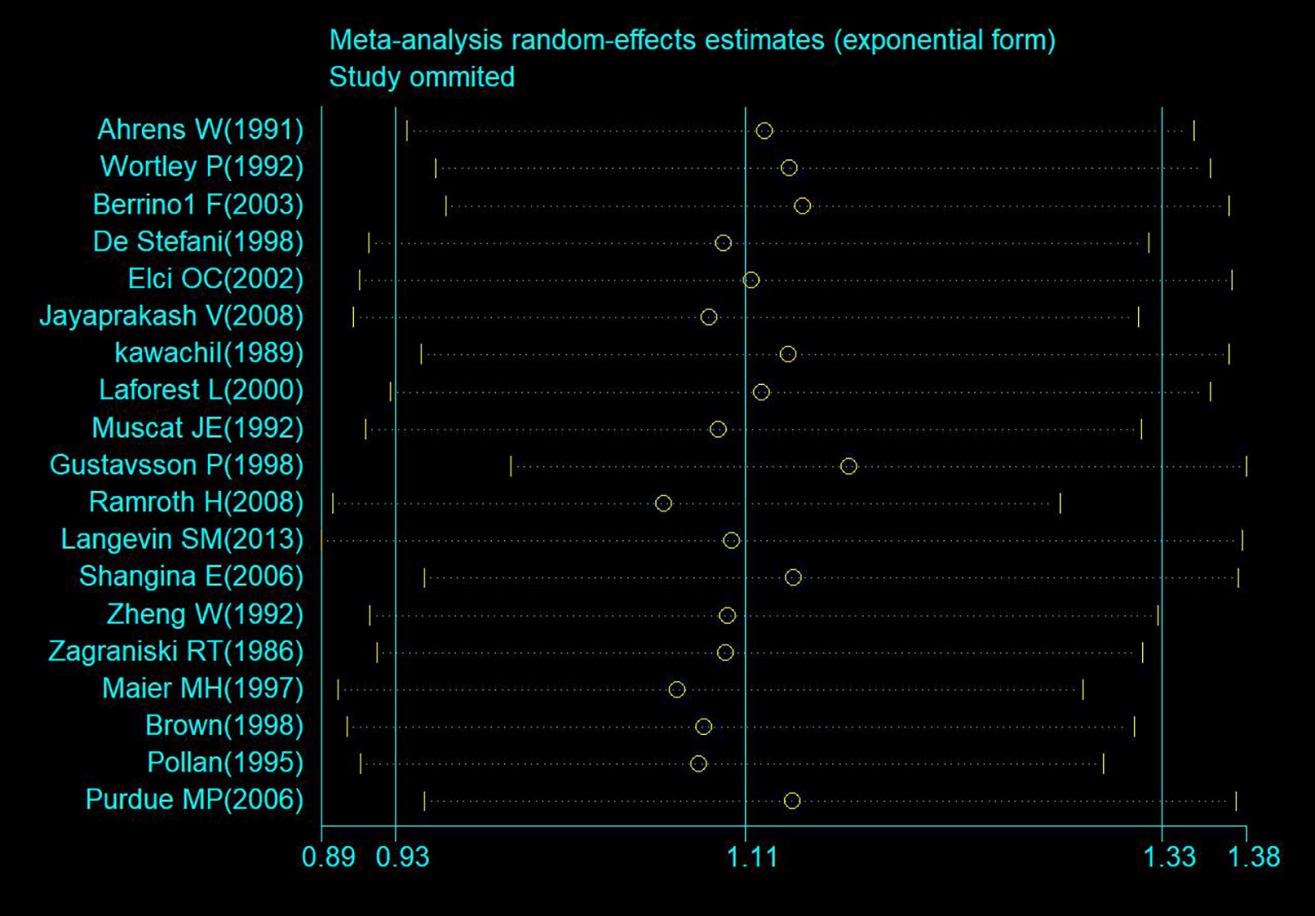
Sensitivity analysis for meta‐analysis of the relationship between occupational/environmental exposure to wood dust and laryngeal cancer.

Nineteen studies (18 case–control studies and one cohort study) assessing the association between wood dust exposure and laryngeal cancer were included in the primary meta‐analysis (Figure 2). On the basis of the collective study findings, wood dust exposure was associated with elevated laryngeal cancer risk, but the association was not statistically significant (OR: 1.11; 95% CI: 0.931.33 [*n* = 19]), and moderate heterogeneity was identified (*I* ^2^ = 45.3%; *p* = 0.017; Figure 2). Sensitivity analyses indicated no change in the combined OR in the leave‐one‐out analysis; the OR ranged from 1.07 (95% CI: 0.90–1.28) to 1.17 (95% CI: 0.99–1.38; Figure 3).

### 3.3 Subgroup Analyses

Wood dust exposure was found to significantly contribute to laryngeal cancer in subgroup analyses based on studies with ≥ 200 cases (OR: 1.14; 95% CI: 1.01–1.25; *I* ^2^ = 41.1%; *p* = 0.083 [*n* = 10]) or < 200 cases (OR: 1.29; 95% CI: 1.00–1.66; *I* ^2^ = 52.0%; *p* = 0.03 [*n* = 9]). A subgroup analysis of wood dust exposure and laryngeal cancer indicated significant associations according to a geographic location in the US (OR: 1.21; 95% CI: 1.07–1.37; *I* ^2^ = 17.1%; *p* = 0.306 [*n* = 5]) or Europe (OR: 1.05; 95% CI: 0.90–1.22; *I* ^*2*^ = *53.2*%; *p* = 0.011 [*n* = 13]).

On the basis of follow‐up evaluations, wood dust exposure led to laryngeal cancer after ≥ 5 years (OR: 1.19; 95% CI: 1.07–1.32, *I* ^2^ = 8.4%; *p* = 0.365 [*n* = 10]) or < 5 years (OR: 1.19; 95% CI: 1.07–1.32; *I* ^2^ = 56.4%; *p* = 0.014 [*n* = 9]). Wood dust exposure was associated with laryngeal cancer, on the basis of studies published after the year 2000 (OR: 1.15; 95% CI: 1.04–1.28; *I* ^2^ = 41.2%; *p* = 0.104 [*n* = 8]) or before the year 2000 (OR: 1.12; 95% CI: 0.89–1.42, *I* ^2^ = 52.5%; *p* = 0.022 [*n* = 11]), as shown in Table 2.

**TABLE 2:** Subgroup analysis of meta‐analysis on the association between occupational/environmental wood dust and laryngeal cancer.

Case	Stduy	OR	95% CI	*I* ^2^	*p*
< 200	9	1.29	1.00–1.66	52.0	0.034
≥ 200	10	1.14	1.01–1.25	41.1	0.083
*District*					
European	13	1.05	0.90–1.22	53.2	0.011
USA	5	1.21	1.07–1.37	17.1	0.306
*Follow year*					
< 5	9	0.97	0.77–1.21	56.4	0.014
≥ 5	10	1.19	1.07–1.32	8.4	0.365
*Published in 2000*					
Before	11	1.12	0.89–1.40	52.5	0.022
After	8	1.15	1.04–1.28	41.2	0.104

### 3.4 Publication Bias

**FIGURE 4:**
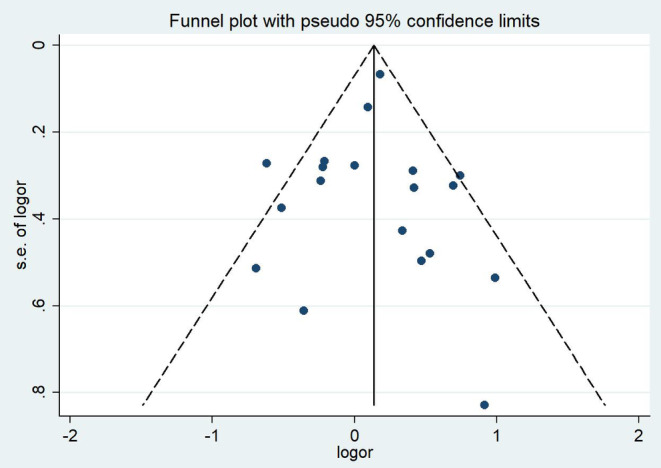
Funnel plot for meta‐analysis of the relationship between occupational/environmental exposure to wood dust and laryngeal cancer.

No evidence of publication bias was detected in funnel plots. Moreover, no evidence of study bias was identified with Begg's test (*p* = 0.675) or Egger's test (*p* = 0.843), as shown in Figure 4.

## 4 Discussion

The upper respiratory and digestive tracts are the main pathways through which dust carcinogens enter the human body. Dust can accumulate in the nasal cavity, larynx, and respiratory tract. The location of deposition depends primarily on the dust particle size, shape, and airflow intensity. Dust deposition causes inflammatory responses and acquired respiratory diseases, chronic inflammation, airway obstruction, and chronic obstructive pulmonary disease.

According to the IARC, wood dust is carcinogenic to humans (group 1) and has a definite role in the development of sinonasal cavity tumors [37]. The role of wood dust in the development of cancer has been a subject of substantial research and speculation. A previous study has reported that workers with occupational exposure to wood dust face an elevated risk of nasopharyngeal cancer [38]. Our previous meta‐analysis [11] has confirmed that wood dust is associated with nasopharyngeal carcinoma [39]. However, studies exploring the relationship between exposure to wood dust and laryngeal cancer occurrence have yielded inconsistent findings.

The pooled results (OR: 0.95; 95% CI: 0.80–1.17; *I* ^2^ = 48.8%) of a recent meta‐analysis have indicated an association between wood dust exposure and laryngeal cancer risk [22], but the populations in Wortley's et al. 1992 study [24] and Vaughan and Davis 1991 study [32] were from the same case–control study, and the associations investigated in Demers's et al. 1995 study [40], Innos's et al. 2000 study [41], and Laakonen's 2006 study [42] were based on the standardized incidence or standardized mortality ratio. Therefore, the current meta‐analysis included only studies reporting results from nonreplicated studies. Laryngeal cancer risk was elevated by wood dust exposure, on the basis of the current meta‐analysis (OR: 1.11; 95% CI: 0.93–1.33 [*n* = 19]), but mild heterogeneity was identified (*I* ^2^ = 45.3%). To ascertain the sources of heterogeneity, we conducted subgroup analyses on the retrieved studies, which indicated that wood dust exposure increased the risk of laryngeal cancer when a larger number of laryngeal cancer cases were present. The most recent report from the IARC has estimated 184,615 new cases of laryngeal cancer and 99,840 deaths worldwide in 2020 [43]; a larger sample size would make the results more significant and decrease the heterogeneity (*I* ^2^ = 41.1% < 45.3%). Given the difference in the predominant wood used between Europe and the US, we performed a subgroup analysis by the geographic region. We observed that wood dust exposure was associated with a significantly elevated risk of developing laryngeal cancer in the US, with decreased heterogeneity (*I* ^2^ = 17.1% < 45.3%). In contrast, among the studies in European countries (particularly Nordic countries), wood dust exposure was not significantly associated with laryngeal cancer. The results by geographic region were consistent with the association between wood dust exposure and lung cancer [44]. Other explanations may exist for the geographic regional differences, including unknown confounding factors and genetic profiles, different occupational and health standards, and levels of health care. Differences in risk estimates might be due to genetic, environmental, or regional differences in health policies. Some European countries are leaders in occupational health and safety practices [45, 46]. To our knowledge, this study reports the first systematic review or meta‐analysis assessing wood dust and laryngeal cancer risk.

Case–control studies are effective in identifying and quantifying occupational exposure as a risk factor for cancer. However, the amount of wood dust exposure depends on factors such as the type of wood (hardwood or softwood), wood‐processing operations (e.g., sawing, milling, grinding, and sanding), processing (e.g., machine and hand sanding), and wood dryness. Workplace size, ventilation, cleaning, and other workplace features might also affect the levels of wood dust exposure. Herein, we focused on studies assessing wood dust exposure, to provide the most direct assessment of the relationship between wood dust exposure and laryngeal cancer risk. We avoided the effects of wood dust due to occupational classification or type of the wood, in agreement with several studies indicating high variation among individual risk estimates when multiple different occupations associated with wood dust exposure are analyzed in the same study [20, 22]. This further adds to the association between wood dust exposure and laryngeal cancer and suggests that our focus on wood dust‐specific studies did not bias the results of this meta‐analysis.

Several limitations of this study must be noted. First, wood dust exposure might not have been assessed accurately because the studies used questionnaires to assess wood dust exposure, but self‐reporting could easily lead to reporting bias. Second, given the complex etiology of laryngeal cancer, the influence of other possible carcinogens present in the workplace (e.g., solvents, asbestos, or formaldehyde) should be considered in addition to factors such as smoking and age.

## 5 Conclusions

Despite these limitations, the findings of the current meta‐analysis suggest that wood dust exposure in the workplace might increase the risk of laryngeal cancer. This meta‐analysis showed an association between wood dust exposure and laryngeal cancer, on the basis of moderate evidence, and a relatively higher risk of laryngeal cancer was observed in studies conducted in the US. Moreover, long‐term exposure and larger sample sizes are necessary to provide sufficient evidence to confirm that wood dust exposure is an independent additional risk factor for laryngeal cancer.

## Registration and Protocol

The protocol is described in the Section 2. Registration does not apply.

## Author Contributions

**E. Meng:** formal analysis (equal), writing – review and editing (equal). **Zhou Xin:** methodology (equal), project administration (equal). **Dou Jianrui:** conceptualization (equal), data curation (equal). **Yin Jinzhu:** data curation (equal).

## Ethics Statement

This article did not involve human or animal research.

## Consent

This article did not involve enrollment of human participants.

## Conflicts of Interest

The authors declare no conflicts of interest.

## Data Availability

The authors have nothing to report.
